# Maintaining relevance in HIV systematic reviews: an evaluation of Cochrane reviews

**DOI:** 10.1186/s13643-019-0960-5

**Published:** 2019-02-07

**Authors:** Ingrid Eshun-Wilson, Shahista Jaffer, Rhodine Smith, Samuel Johnson, Paul Hine, Alberto Mateo, Anne-Marie Stephani, Paul Garner

**Affiliations:** 1 Center for Evidence Based Health Care, Division of Epidemiology and Biostatistics, Department of Global Health, Stellenbosch University, Francie Van Zyl drive, Cape Town, 7505 South Africa; 2 Cochrane Infectious Diseases Group, Centre for Evidence Synthesis in Global Health, Liverpool School of Tropical Medicine, Liverpool, UK; 3 University of Central Lancashire, Lancashire, UK; 4 University of California, San Francisco, USA

## Abstract

**Background:**

Research turnover in the HIV field is rapid, and as a result, maintaining high-quality, up-to-date, and relevant systematic reviews is a challenge. One approach is to frequently update published reviews.

**Methods:**

We evaluated the methods and relevance of all HIV systematic reviews and protocols published in the Cochrane Library over a 16-year period (2000–2016) to determine the need to update published reviews or complete of reviews in progress.

**Results:**

Of 148 published reviews and protocols, 129 (87%) were identified as not for updating or progression to publication, mostly due to research questions which were either entirely outdated or addressed questions in an outdated manner (*N* = 89; 60%); this was anticipated for older reviews, but was found also to be the case for recent publications. Some research questions were also inadequately conceptualized, particularly when complex pragmatic trials or behavioral interventions were included.

**Conclusions:**

We suggest that authors clearly characterize interventions and synthesis approaches in their review protocols. In research fields, such as HIV, where questions change frequently, systematic reviews and protocols should be regularly re-evaluated to ensure relevance to current questions. This process of re-evaluation should be incorporated into the methods of living systematic reviews.

**Electronic supplementary material:**

The online version of this article (10.1186/s13643-019-0960-5) contains supplementary material, which is available to authorized users.

## Background

The HIV field has high research outputs, and the cycle of questions, trials, and guideline modification is rapid. This raises the question of how to keep HIV systematic reviews up to date, relevant, and derived from the highest methodological standards [1, 2]. One approach is to regularly update reviews to incorporate new findings; and indeed, some questions endure and should be updated; and for others however, the review question may have been answered, changed substantially, or become irrelevant, and blindly updating such reviews can result in wasted effort and resources. To provide insight into how review questions change over time, we formally appraised the relevance, methods, and need to update a suite of HIV systematic reviews and protocols published in the Cochrane Library between 2000 and 2016.

## Methods

Between 1 May 2016 and 30 May 2018, we critically appraised all published Cochrane HIV reviews and protocols based on the Cochrane Updating Classification System [3, 4]. The Updating Classification System acts as a guide to readers to assess whether a review is up to date, needs updating, or might need updating in the future [3, 4]. We modified the tool for assessing protocols. Reviews and protocols were appraised by evaluating: (a) the currency and priority of the review question (through discussion with experts in the field and exploring the literature), (b) the methods, by using an abbreviated version of the MECIR standards [2], and (c) whether there were new studies to be included in the review, or whether another high-quality review had already been published on the topic outside of Cochrane. Based on the outcome of this assessment, we determined whether reviews should be updated, and whether protocols should continue to full reviews. We categorized them as follows: (1) continue with review update or allow protocol to progress to full review and (2) not for update or protocol progression due to (a) methodological concerns, (b) outdated research question, or (c) review already up to date. At least two people read and appraised each review independently, and the classification was agreed in team meetings chaired by experienced systematic review authors. The tool is available in the Additional file 1.

## Results

We identified and appraised 109 Cochrane HIV reviews and 39 HIV protocols (148 articles in total), published on the Cochrane Database of Systematic Reviews between 2000 and 2016. Review and protocol topics included questions about HIV treatment interventions (63%), HIV prevention interventions (32%), health system interventions (2%), and interventions to improve HIV diagnosis (3%). The majority of reviews (*N* = 98; 66%) were published between 2010 and 2016, with a smaller proportion published between 2000 and 2009 (*N* = 50; 34%). We concluded that 19 (13%) of all reviews and protocols were eligible for an update or progression to full review, and the remaining 129 (87%) were assessed as “not for updating or progression”.

**Fig. 1:**
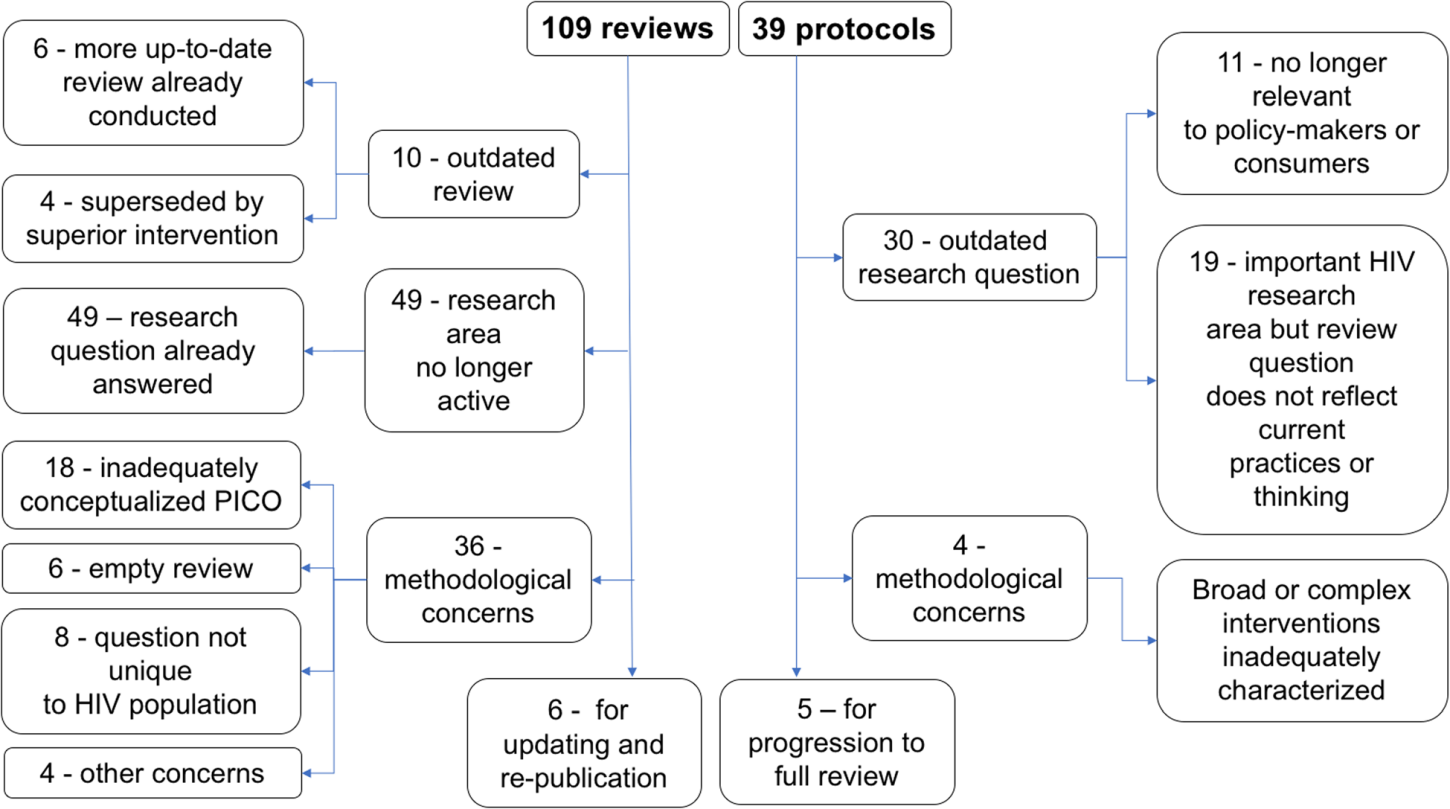
Outcomes of review and protocol appraisals

Of the 109 published systematic reviews, 95 were assessed as “not for updating” (87%). Of these, 62% had outdated research questions (Fig. 1) where the intervention was superseded by a superior intervention or by another more up-to-date review, or where questions were already answered, and there was little to no ongoing research in the topic area. For example, a review of the “Effectiveness of first-line tenofovir, emtricitabine and efavirenz for patients with HIV” would not require updating as this question has been answered. Another example is the use of HSV-2 suppressive therapy for HIV prevention: this is no longer considered an option for HIV prevention; although there may be some additional studies for inclusion, updating such a review would be of little value to consumers or policy makers. We discontinued 11 reviews due to methodological errors (37%): this was the result of inadequately conceptualized research questions (the population, interventions, comparison, or outcomes were not sufficiently characterized or relevant). Sometimes, this led to “empty” reviews (with no included studies after searches were conducted). Another methodological problem was review questions which were not unique to HIV populations, for example, reviews evaluating drug effects in HIV subpopulations with no clear rationale for why these agents would have different outcomes in HIV-positive people.

Thirty-four of 39 protocols were stopped from progressing to full reviews (87%) (Fig. 1). The two main reasons for discontinuation also included outdated research questions. This included protocols where the topic was no longer relevant to policy makers or consumers, or where an important HIV research area was evaluated but the protocol review question did not reflect current practices or thinking. The remaining protocols were discontinued due to methodological limitations, as a result of inadequately conceptualized research questions. Specifically, protocols encompassed a very broad set of treatment strategies that were difficult to combine in one review, or authors did not consider how to meaningfully combine complex HIV interventions. This was common in protocols which included behavioral HIV prevention interventions and pragmatic implementation trials. Thirty protocols were published more than 2 years previously (interquartile range 3–9 years).

## Discussion

This appraisal of Cochrane systematic reviews and protocols demonstrates that topics in the HIV field become outdated quickly, and this was by far the commonest reason for discontinuation of both reviews and protocols, including those from more recent years. In addition, changes in reporting standards over time resulted in outdated methodology. Protocols which progressed slowly became irrelevant before completion, and inadequately conceptualized research questions resulted in disorganized or empty reviews which, at times, did not address issues specifically relevant to HIV populations, or sufficiently characterize interventions and key HIV outcomes.

Evaluating the relevance of systematic reviews prior to updating is essential in all fields but particularly important where there is rapid development of new innovations and care strategies, as seen in HIV research. Where there are delays, protocols should be re-evaluated and adapted to ensure that final reviews answer current questions. With the recent drive to automate and create “living” systematic reviews with frequent updates [5, 6], it is essential that methods of maintaining currency are incorporated into these evidence synthesis approaches as questions may change or become irrelevant [7, 8].

We recommend that authors planning to conduct HIV systematic reviews reflect on how their review can contribute, by thoroughly exploring the current evidence, determining if primary studies exist on the topic of interest, identifying other systematic reviews already conducted, and clearly defining their research question and what their review may add. In addition to presenting the PICO (population, intervention, comparison, and outcomes) elements, authors should fully characterize the interventions of interest, limit the number and types of interventions, and determine how to synthesize results from studies with behavioral or complex interventions. Several available tools can be drawn on and adapted to aid the design of conceptual frameworks and characterization of interventions and complexity: this can help structure review questions to produce meaningful results [7–11]. Review team effort and Cochrane editorial procedures need to ensure timely completion of reviews to avoid them being outdated before completion.

In summary, HIV research questions and the systematic reviews summarizing the relevant research evidence become outdated quickly, and rapid conduct and publication is critical; clearly defined research questions need to be regularly re-evaluated alongside changes in the field to maintain relevance, particularly in the era of living systematic reviews.

## Additional files

Additional file 1: Modified tool for assessing reviews and protocols. (DOCX 18 kb)

## Abbreviations

HIV: Human immunodeficiency virus
HSV-2: Herpes simplex virus 2
IQR: Interquartile range
MECIR: Methodological Expectations of Cochrane Intervention Reviews
