# Supplementary material for: Maintaining relevance in HIV systematic reviews: an evaluation of Cochrane reviews
Source: Syst Rev. 2019 Feb 7;8:46. doi: 10.1186/s13643-019-0960-5 (PMC6366015; doi:10.1186/s13643-019-0960-5)
Supplement: Supplementary file 1 — Modified tool for assessing reviews and protocols. (DOCX 18 kb) [file 13643_2019_960_MOESM1_ESM.docx]

| 1. **Quality Assessment** | | | | | | |
| --- | --- | --- | --- | --- | --- | --- |
| **MECIR Standard** | | | | **Included (Y/N)?** | **To the required standard (Y/N)?** | |
| **C1** | Formulated review question | | |  |  | |
| **C5** | Pre-defined inclusion criteria | | |  |  | |
| **C44** | Description of included studies/interventions | | |  |  | |
| **C52** | Risk of Bias Assessment | | |  |  | |
| **C75** | Summary of Findings table | | |  |  | |
| **2. Editorial assessment** | | | | | | |
|  | **1** | **2** | **3** | **4** | **Score** | |
| **Is the review approach appropriate to the question?** | No, likely to require a revision of the protocol | No, likely to need substantial re-structuring | Yes, likely to need only minor re-arrangement | Yes, ready for copy-editing |  | |
| **Is the writing of adequate scientific quality?** | No, major scientific flaws and poor quality writing.  Would be rejected by any major journal | No, scientifically sound but needs to be re-written in appropriate language | Yes, requires only minimal editorial input and copy-editing | Yes, ready for copy-editing |  | |
| **Will the review require substantial editing for length?** | Yes, multiple major sections are overwritten and much too long | Yes, at least one major section is much too long and needs re-writing | No, requires only minimal editing for length | No, ready for copy-editing |  | |
| **CIDG support required to meet quality standards** | Requires major CIDG input into concept and structure, and major revision | Requires detailed CIDG feedback and major revision | Suitable for editor review with minor revisions and minimal CIDG input | Ready for copy-editing |  | |
| **3. Importance of the question** | | | | | | |
|  | **1** | **2** | **3** | **4** | **Score and justification** | |
| **Assessment** | Not important.  The question is either historical or if minimal importance. | Of limited interest.  Academic interest only, unlikely to influence policy or be of interest to consumers | Moderate interest.  To policy makers and consumers | Important current question | Content editor: |  |
|  |  |  |  |  | Consumer: |  |
|  |  |  |  |  | Co-ordinating Editor: |  |
| **4. Overall assessment** | | | | | | |
|  | | **Quality** | | Recommendation: | Comment: | |
|  |  | High | Low |  |  | |
| **Priority** | HIgh |  |  |  |  |  |
|  | Low |  |  |  |  |  |
| **5. New research** | | | | | | |
| **Any new primary studies or systematic reviews relevant to decision?** [Y/N] | | | What are the studies and how are they relevant: | |  | |
| **6. Recommendations** | | | | | | |
| **Recommendation:** | **Reason:** | | **Summary justification:** | | | |
| **No update planned** | Intervention/index test superseded | |  | | | |
|  | Invalid methods/poorly conducted | |  |  |  |  |
|  | Review superseded | |  |  |  |  |
|  | Research area no longer active | |  |  |  |  |
|  | Low impact usage | |  |  |  |  |
|  | Other | |  |  |  |  |
| **Up-to-date** | No new studies identified with search | |  | | | |
|  | All studies incorporated from most recent search | |  |  |  |  |
|  | Relevant studies ongoing but not completed | |  |  |  |  |
|  | Certainty/quality high in published review | |  |  |  |  |
|  | New info but unlikely to change review findings | |  |  |  |  |
| **Update recommended** | Authors currently updating | |  | | | |
|  | Studies awaiting assessment | |  |  |  |  |
|  | New contributors needed | |  |  |  |  |
|  | Other | |  |  |  |  |
